# Dutch and English toddlers' use of linguistic cues in predicting upcoming turn transitions

**DOI:** 10.3389/fpsyg.2015.00495

**Published:** 2015-04-24

**Authors:** Imme Lammertink, Marisa Casillas, Titia Benders, Brechtje Post, Paula Fikkert

**Affiliations:** ^1^Centre for Language Studies, Radboud UniversityNijmegen, Netherlands; ^2^Language and Cognition Department, Max Planck Institute for PsycholinguisticsNijmegen, Netherlands; ^3^School of Psychology, Newcastle UniversityNewcastle, NSW, Australia; ^4^Department of Theoretical and Applied Linguistics, Cambridge UniversityCambridge, UK

**Keywords:** turn prediction, prosody, lexicosyntax, child language, eye-tracking

## Abstract

Adults achieve successful coordination during conversation by using prosodic and lexicosyntactic cues to predict upcoming changes in speakership. We examined the relative weight of these linguistic cues in the prediction of upcoming turn structure by toddlers learning Dutch (Experiment 1; *N* = 21) and British English (Experiment 2; *N* = 20) and adult control participants (Dutch: *N* = 16; English: *N* = 20). We tracked participants' anticipatory eye movements as they watched videos of dyadic puppet conversation. We controlled the prosodic and lexicosyntactic cues to turn completion for a subset of the utterances in each conversation to create four types of target utterances (*fully incomplete, incomplete syntax, incomplete prosody*, and *fully complete*). All participants (Dutch and English toddlers and adults) used both prosodic and lexicosyntactic cues to anticipate upcoming speaker changes, but weighed lexicosyntactic cues over prosodic ones when the two were pitted against each other. The results suggest that Dutch and English toddlers are already nearly adult-like in their use of prosodic and lexicosyntactic cues in anticipating upcoming turn transitions.

## Introduction

Speakers in conversation take turns at talking (Sacks et al., 1974). The timing of speaker transitions is precise, usually exhibiting a 200 ms gap or a brief period of vocal overlap between turns (Stivers et al., 2009). Considering that it takes approximately 600 ms to initiate speech production (based on object naming; Levelt, 1989), addressees must anticipate when the current speaker's turn will end and must start planning their response well in advance to achieve minimal gap and minimal overlap transition timing (Levinson, 2013). This process requires the addressee to perform multiple tasks at once—decoding and interpreting the speech signal, plus formulating and articulating an appropriate response—all within the last few syllables of the ongoing turn (Levinson, 2013). Children, whose linguistic skills are still developing, have a hard time accomplishing these multiple tasks for turn-taking; it takes them several years before they master adult-like turn-taking behavior (age 6; Casillas et al., in press; Ervin-Tripp, 1979; Garvey, 1984).

Despite their late mastery of turn-taking, children begin taking turns (of a sort) in infancy. Caregivers respond to their 3–4-month-old infants' vocalizations, movements, and vegetative sounds as if they were “turns” in proto conversation (Bruner, 1975; Snow, 1977; Ginsburg and Kilbourne, 1988). Twelve-month-olds already understand conversational patterns well enough to expect speech (but not non-speech) sounds to provoke a verbal response from an addressee (Thorgrímsson et al., 2015). One- and two-year-olds watching videos of conversation look anticipatorily to the upcoming responder at points of turn transition (Casillas and Frank, 2013). However, in their spontaneous turn-taking behavior, and in their predictions about upcoming turn boundaries, children are generally slower and less accurate compared to adults.

To anticipate upcoming turn structure accurately, children must learn to use predictive information in the ongoing speech signal. Recent experimental findings demonstrate that toddlers use both lexicosyntactic and prosodic information to predict upcoming speaker switches, but the relative importance of these information sources for prediction remains largely undetermined (Casillas and Frank, 2012, 2013; Keitel et al., 2013; Keitel and Daum, 2015). The current study investigates how Dutch and English toddlers weigh lexicosyntactic and prosodic^1^ cues against one another in their online prediction of upcoming speaker switch.

Lexicosyntactic cues can provide critical information about upcoming speaker switches. For example, incomplete syntactic structures (“I'm making the…”) hint that there is still more information to come, frequent multi-word sequences or strong semantic associations between words (“I need to brush my…”) can strongly indicate what exact words will come next, and the word order of an utterance (interrogative vs. declarative) can help listeners predict how the current turn will finish (and what will happen in the next turn). Lexicosyntactic information appears to be critical for adult turn-end prediction: listeners anticipate turn-end timing more accurately when they can predict the exact words that will make up the rest of the turn (Magyari and de Ruiter, 2012). Speaker changes also almost always occur at points of lexicosyntactic completion in task-oriented dialogs (Dutch: Caspers, 2003; English: Ford and Thompson, 1996), and lexical and syntactic cues to questionhood (e.g., *wh*-words and subject-auxiliary inversion) occur early in the turn, thereby giving addressees more time to begin planning their response early (Bögels et al., 2014).

At least one previous study suggests that lexicosyntactic information is more important than prosodic information in adults' predictions about upcoming speaker changes. de Ruiter et al. (2006) asked participants to listen to fragments of speech and to press a button when they felt that the speaker's turn was coming to an end. Listeners achieved the same button press accuracy for normal speech (with full linguistic information) and intonationally flattened speech (with lexicosyntax, rhythm, and intensity, but no intonational information). In contrast, participants' accuracy significantly decreased for low-pass filtered speech (with full prosodic cues, but no lexicosyntactic information). The authors took this result as evidence that lexicosyntactic cues are primary, and possibly sufficient, for adult turn prediction, while prosodic cues play a less important role.

Other work has characterized lexicosyntactic and prosodic cues as having qualitatively different functions for turn prediction. Under this view, lexicosyntax is particularly important in assessing whether a turn is complete and, by extension, whether it is ripe for a speaker switch. In natural speech, Dutch and English listeners rarely expect speaker switches when lexicosyntactic information is incomplete, no matter what intonational contour is used (Caspers, 2001; Wichmann and Caspers, 2001). But, when speakers have multi-utterance turns, and the addressee has to pass over several lexicosyntactically complete phrases before reaching the true turn-end, lexicosyntax alone does not provide sufficient information. Then prosody plays a critical role in listeners' ability to discriminate between *potential* completion points and *true* completion points. Turn-ends are often accompanied by prosodic cues such as boundary tones, increased syllable length, and post-turn silence (Ford and Thompson, 1996). Whether listeners expect a speaker change at lexicosyntactically complete points is largely dependent on the prosodic cues in the utterance (Caspers, 2001; Wichmann and Caspers, 2001).

The present paper addresses how children learn to use lexicosyntactic and prosodic cues in their prediction of upcoming turn structure. Generally speaking, children are sensitive to prosodic information before they become sensitive to lexicosyntactic information. Newborn infants use prosodic cues to distinguish their native language from other languages (Nazzi et al., 1998). Seven-month-olds can also use prosodic information to distinguish between words spoken with an angry, happy, or neutral voice (Grossmann et al., 2005). By 10 months, they can also use prosodic cues to segment the speech stream into smaller units (Gleitman and Wanner, 1982; Jusczyk, 1997; Christophe et al., 2008).

It is often assumed that children's sensitivity to prosodic information bootstraps their sensitivity to lexicosyntactic information (Morgan and Demuth, 1996; Christophe et al., 2008; Männel and Friederici, 2010). Newborns can discriminate categories of function words and content words on the basis of their different prosodic characteristics (Shi et al., 1999). Children show sensitivity to the word order of their native language as young as 7–8 months of age on the basis of word frequency and prosody (Höhle and Weissenborn, 2003; Gervain and Werker, 2013). Once children's knowledge of lexicosyntactic information becomes more detailed, they can access lexical and syntactic structures independently from the prosodic information available. For example, children start to recognize distinct function words at 11 months of age (Shi et al., 2006) and children at 12 months of age can use differences in word order to distinguish between questions and declaratives (Geffen and Mintz, 2014).

Given that sensitivity to prosodic cues precedes, or even bootstraps, sensitivity to lexicosyntactic cues, prosodic cues might have an early and primary role in children's predictions about upcoming speaker change. But recent studies have only found mixed evidence for this hypothesis. Casillas and Frank (2013) showed videos of conversation to children and adults, finding that children three and younger needed prosodic information to make above-chance anticipatory gaze switches to upcoming speakers in the video. In the same study, children three and older *did* show more gaze switches for lexical-only stimuli than for prosody-only stimuli, but only for question-answer speaker switches: in conditions where lexical information was available, children made more anticipatory gaze switches after hearing questions than non-questions. Their results suggest an early, more global role of prosody in turn prediction and a later, question-specific role of lexicosyntax. Importantly, the stimuli in their experiment were phonetically manipulated to control for linguistic information, e.g., using speech that was low-pass filtered, intonationally flattened, duration-controlled, and multi-layered (but see also Casillas and Frank, 2012). Children do not often hear this kind of phonetically controlled speech in their natural language environment.

Keitel et al. (2013); Keitel and Daum (2015) performed a similar study, showing children videos of conversation and using children's age (rather than phonetic manipulation) to control for the availability of lexicosyntactic cues; they tested both pre-verbal (6- and 12-month-old) and verbal (24- and 36-month-old) children. To test for the role of intonation, half of the videos featured pitch-flattened speech and the other half featured a full linguistic signal. Children only made above-chance anticipatory gaze switches to the upcoming responder at 36 months—considerably later than what Casillas and Frank (2013) found—and anticipated speaker changes less often when intonational contours were removed (but only at 36 months). In contrast, adults' turn predictions were unaffected by the lack of intonational contours. The findings indicate that intonation may be useful for children's turn prediction, but only at age three and up, and not for adults. But, again, the primary linguistic control in the stimuli depended on phonetic manipulation of the speech signal. Thus, the results of these prior studies—Casillas and Frank (2013) and Keitel et al. (2013); Keitel and Daum (2015)—are based on a comparison between natural (full signal) and non-natural (phonetically manipulated) stimuli.

Unnatural speech is noticeable to children, and more generally changes the way listeners process linguistic information. Twelve- and 36-month-olds prefer speech sounds to non-speech (motor) sounds while watching videos of conversation (Bakker et al., 2011). If children in the studies mentioned above interpreted the manipulated speech as degraded or even as non-speech, they might have processed the lexicosyntactic and prosodic information differently than they do in everyday interactions. Even for adults, acoustically unusual stimuli, such as synthetic speech, can cause significant processing costs (Pisoni, 1981).

The current study is designed to assess the relative and the individual contributions of prosody and lexicosyntax for turn structure prediction while using the full speech signal (unfiltered speech with both lexicosyntactic and prosodic cues present). We used a full speech signal so that we could test children's use of linguistic cues for speaker-switch prediction with stimuli that resemble speech in their natural environment—stimuli without any phonetic filtering or resynthesis. Participants watched eight videos of short, scripted conversation. For a subset of the utterances in each conversation, we controlled for the presence of lexicosyntactic and prosodic cues to turn completion by cross-splicing snippets from multiple sentence recordings (see Section Stimulus Preparation). In one condition, both lexicosyntax and prosody signaled an upcoming speaker switch (a fully complete turn). In the opposite condition, neither cue signaled an upcoming speaker switch (a fully incomplete turn). In two more conditions, lexicosyntax, and prosody were pitted against each other to test for their relative primacy (i.e., complete lexicosyntax with incomplete prosody *or* incomplete lexicosyntax with complete prosody). We expected that young children would rely more on prosodic cues in their prediction of upcoming turn structure, given their early acquisition of basic prosodic knowledge.

Following recent work, we measured children's predictions about upcoming turn structure by tracking their eye movements while they watched videos of dyadic conversation between puppets. In line with previous studies investigating children's anticipation of turn structure (Casillas and Frank, 2013; Keitel and Daum, 2015), we used puppet dyads to capture children's attention while also conveniently removing the non-verbal cues to turn taking that often appear at turn boundaries (e.g., gaze and gesture; Rossano et al., 2009; Stivers and Rossano, 2010). The absence of non-verbal cues enabled us to focus on the role of linguistic cues.

Eye tracking is a natural and passive measure of attention, but provides an online measure of children's predictive processing during conversation (Casillas and Frank, 2012, 2013; Keitel et al., 2013; Keitel and Daum, 2015). Prior work has shown that, compared to explicit measures of turn-end prediction (e.g., button-press; de Ruiter et al., 2006), anticipatory eye movements from the prior to the next speaker tend to occur quite late at points of speaker transition (children: Casillas and Frank, 2012, 2013; Keitel et al., 2013; Keitel and Daum, 2015; and adults: Tice and Henetz, 2011; Hirvenkari et al., 2013; but also see Holler and Kendrick, 2015 for earlier switching in adults). Eye-tracking measures therefore do not target turn-end prediction the same way that button press measures do. Instead, they appear to index the prediction of upcoming turn transitions and the onset of an upcoming response, both of which are affected by linguistic material present in the pre-transition turn (e.g., question vs. non-question, prosodic information, etc…). We track participants' anticipatory eye movements to upcoming speakers as a natural measure of their predictions about upcoming turn structure.

We sampled from two linguistic populations to test the robustness of our findings: Dutch (Experiment 1) and British English (Experiment 2). Dutch and English use similar linguistic structures to form simple declaratives and polar interrogatives; in both languages the subject precedes the verb in declarative utterances, whereas interrogative utterances are created by subject-verb inversion (Dryer, 2013). The prototypical intonation pattern for polar questions in both languages also features a final rise^2^ (e.g., Dutch: Haan, 2002; English: Grabe and Post, 2004).

## Experiment 1

### Materials and methods

#### Participants

Thirty-three native Dutch-speaking 2.5-year-olds participated in the experiment. Of these, twelve were excluded because of equipment error (1) or inattention to the screen during the experiment (11; see Section Data Pre-Processing). As a result, 21 toddlers were included in the final analysis (Female = 13, mean age = 29 months, range = 24–33 months). Sixteen adult participants (native Dutch speakers, Female = 15, mean age = 23 years) participated as a control group. No hearing or vision problems were reported. Ethical approval for the study was obtained from the *Ethiek commissie faculteit der Sociale Wetenschappen (ECSW)* at Radboud University in Nijmegen.

#### Apparatus

We recruited and tested toddlers through the Baby Research Center (BRC) in Nijmegen, The Netherlands. The data were obtained with a 17-inch Tobii 1750 eye-tracker (Tobii Technology AB; binocular infrared light reflection, 50 Hz sampling frequency, accuracy range: 0.5° to 1°, recovery <100 ms). Eye-tracker calibration and stimulus presentation were controlled by ClearView 2.7.1 software. Audio speakers were placed at either side of the screen, hidden from participant view. Participants sat approximately 60 cm from the monitor, with toddlers sitting on their parent's lap.

#### Procedure

Each session began with a 9-point infant-friendly calibration procedure. Data collection started when good calibration for both eyes was obtained for at least five locations on the screen (every corner and the center). Children then watched eight 30-s videos of conversation between two puppets. Before each conversation, the experimenter displayed an animated smiley face on the screen until children's gaze returned to the center. After every two conversations, participants saw a 4–9-s animated filler video (a train, a skating dog, and a running chick). The experiment took 5 min in total. Two versions of the experiment were created, with conversation videos ordered differently in each. In both versions, the same pair of puppets was shown, at most, twice in a row. Participants were randomly assigned to one of the two versions.

#### Audio stimuli

#### Target utterances

We created four types of target utterances by controlling for lexicosyntactic and prosodic cues to turn completion (Table 1). At the point of syntactic completion (or incompletion) for each target utterance we inserted 500 ms of silence (“[…]” in Table 1). Participants could then make a prediction, depending on the linguistic information up to that point, about whether the same speaker would continue or whether the addressee would respond. We measured participants' anticipatory gaze to the addressee around these 500 ms silent windows.

**Table 1:** **(A) Examples of target utterances in the four conditions. (B) Example of a conversation with the four target utterances embedded in six filler utterances**.

**(A) Target utterances**			**(B) Conversation**	
**Condition**	**Cues**	**Example**	**Speaker**	**Example**
			A	I think I'll go swimming today
(1) Fully incomplete	−SYN	Today is a beautiful+ […] +day for a swim	B (1)	Today is a beautiful+ […] +day for a swim
	−PROS		
			B	And I have a new swimsuit
(2) Incomplete syntax	−SYN	It's made especially for? […] Swimming in the ocean	B (2)	It's made especially for? […] Swimming in the ocean
	+PROS		
			A	Wow, you should try it out then
			A	I bet you bought a really nice one
(3) Incomplete prosody	+SYN	Do you enjoy swimming+ […]	B (3)	Do you enjoy swimming+ […]
	−PROS			
			A	Yes, I like to swim a lot
(4) Fully complete	+SYN	Shall we swim together? […]	B (4)	Shall we swim together? […]
	+PROS			
			B	That would be really fun

*Each utterance is marked as syntactically complete (+SYN) or incomplete (−SYN), and prosodically complete (+PROS) or incomplete (−PROS). The symbol “?” indicates a complete, polar interrogative pitch contour, “+” indicates the lack of a boundary tone, and “[…]” indicates a 500 ms silence. Examples are taken from the British English stimuli used in Experiment 2*.

The utterances with cues to turn completeness featured polar interrogative syntax (+SYN), a polar interrogative pitch contour (+PROS; a high, final rise in Standard Dutch; Haan, 2002), or both. The utterances with cues to turn incompleteness featured incomplete declarative syntax (−SYN), incomplete non-interrogative pitch contours (−PROS), or both. The incomplete non-interrogative pitch contours were deemed “incomplete” because they lacked boundary tones at the onset of the inserted 500 ms silence.

By this design, fully complete utterances were both lexicosyntactically and prosodically complete, and took the form of polar interrogatives with a final rise pitch contour, followed by 500 ms of silence (e.g., *Shall we swim together?* […]). Meanwhile, fully incomplete utterances were both lexicosyntactically and prosodically incomplete at the onset of the 500 ms silence. These fully incomplete utterances took the form of declarative sentences that had been split into two parts by 500 ms of silence; at the onset of the silence (where we measured participants' anticipatory gaze), the in-progress utterance was lexicosyntactically incomplete and had no final boundary tone (e.g., *Today is a beautiful+* [*…*] +*day for a swim*).

The two other target utterance types were only partially complete. For example, utterances that were prosodically complete but lexicosyntactically incomplete took the form of declarative sentences that had been split into two parts by 500 ms of silence; at the onset of the silence, the in-progress utterance was lexicosyntactically incomplete but prosodically complete, with a final rise pitch contour (e.g., *It's made especially for?* […] *swimming in the ocean*). Meanwhile, utterances that were lexicosyntactically complete but prosodically incomplete, took the form of complete polar interrogatives that lacked a final boundary tone at the onset of the 500 ms of silence (e.g., *Do you enjoy swimming*+ […]). Table 1 gives an example conversation that demonstrates the placement of the 500 ms silences for each utterance type.

With this design, all lexicosyntactically complete utterances were interrogative and all lexicosyntactically incomplete utterances were declarative. In designing the utterance types this way, we created a maximal contrast in participants' expectations about an upcoming turn switch between the *fully complete* and *fully incomplete* utterances. Questions naturally project an answer in the next turn, and so observers could reliably expect a turn transition after hearing a question (Casillas and Frank, 2012, 2013). Declaratives do not necessarily project a turn transition, and so observers' expectations after declaratives are much weaker. We sought to create a maximal difference in the fully complete and incomplete conditions because they served as the baselines for our primary conditions of interest: the partially complete conditions (incomplete syntax and incomplete prosody).

We could have instead tried to keep word order the same across the complete and incomplete lexicosyntactic conditions, but this would have created other problems. For example, using interrogative word order for all utterance types would have signaled turn transition early on in the utterance for all sentences, yielding ambiguous and unnatural sentences in the lexicosyntactically incomplete condition (“Would you like a+”). Using declarative word order in both conditions could have possibly worked; declarative polar questions *do* occur in spontaneous Dutch and English (Gunlogson, 2001; Englert, 2010). But declarative polar questions are primarily used for the initiation of repair or for confirmation requests, whereas interrogative polar questions are primarily used for requesting information (Englert, 2010). Thus, even if we used declarative word order for all utterance types, the speech acts would still differ across types. Additionally, to use declarative polar questions, we would need to generate the required contexts for declarative questioning into the scripts (e.g., potential mishearing/misunderstanding), thereby introducing further variation across conversations. Considering these issues together, we decided to use interrogative polar questions for lexicosyntactically complete conditions and unfinished declaratives (at the onset of the 500 ms silence) for lexicosyntactically incomplete conditions. There were eight target sentences in each conversation, resulting in 32 total target sentences. Each of the four conditions for target sentences is described below.

#### Conversation design

The targets were embedded in eight 30-s scripted conversations about topics familiar to 2.5-year-olds (rabbits, snowmen, swimming, birthday parties, and bicycles; Zink and Lejaegere, 2003). Every conversation had six filler and four target utterances, including one target utterance from each type (Table 1B). Targets and filler utterances were separated by 500 ms of silence.

The order of the target utterances within the eight conversations was counterbalanced. Target utterances were equally divided between the two speakers across the eight conversations of the experiment. After *fully incomplete* and *incomplete syntax* target utterances, no turn transition occurred following the 500 ms of silence; the current speaker always completed her turn. After *incomplete prosody* and *fully complete* target utterances, target turns were followed by 500 ms and then a change in speakership 50% of the time. Each conversation contained from five to seven turn transitions.

#### Stimulus preparation

The audio stimuli were recorded in a sound-attenuated booth by two female native speakers of Standard Dutch. The audio for each experiment was collected over two recording sessions. In the first session, both speakers were recorded simultaneously while they acted out the eight dialogs together, three times each. Speakers were asked to read each conversation in an infant-directed register. The filler utterances were then extracted from the best recording of each conversation and then set aside for use in the final stimuli. In the second recording session, speakers were recorded individually as they read an additional set of recording utterances aloud. The additional recording sentences were designed to elicit sub-parts of the target sentences—subparts that could then be spliced together to create the final target utterances (see below). In the second recording, speakers matched their pitch, speaking rate, and affect to the first recording by listening to the first-session conversations over a pair of headphones. The final target utterances were then spliced together from these second-session utterances, and then the conversations were spliced together from a combination of the filler and target utterances.

**Figure 1:**
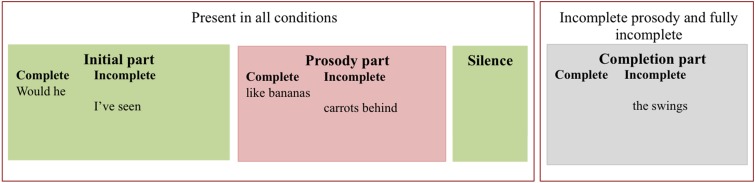
**Division of two target utterances into their subparts**. The initial part (green), the prosody part (red), and the silence (green) were present in all target utterances. The completion part (gray) was only present in lexicosyntactically incomplete target utterances.

We composed each target utterance from three or four parts: an initial part, a prosody part, a silence, and (for lexicosyntactically incomplete utterances) a completion part (Figure 1). Each part derived from a separate recording utterance (from the second recording session). The parts were then spliced together to obtain the final set of target utterances (Praat; Boersma and Weenink, 2012).

The “initial part” of the target utterance was two words long, with an utterance-initial non-interrogative prosodic contour. For example, the “*I've seen*” in “*I've see carrots behind the swings*” was extracted from the recording sentence “***I've seen*** *caramel”* (Figure 1).

The “prosody part” was also two words long. For prosodically incomplete target utterances, there was no prosodic boundary at the end of the second word. For example, “*carrots behind*” was extracted from the recording sentence “*I've seen* ***carrots behind*** *the broccoli for weeks”*. In these recording sentences, the utterance always continued beyond the splicing point to ensure that there was no intonational phrase boundary at the end of the two-word prosodic part. On the other hand, for prosodically complete utterances, the two-word prosody part had a complete, interrogative prosodic contour. For example, “*like bananas”* was extracted from the recording sentence “*Said he:* ‘***like bananas?***’” (Figure 1).

The prosody part was followed by 500 ms of silence. Although 500 ms is somewhat long for an inter-turn gap in adult conversation (Stivers et al., 2009), it closely resembles the median response latency for children in interaction with their parents (549 ms for children's responses at 2;4–2;5; Casillas et al., in press) and it is much shorter than their median response latency with their peers (900 ms for children's responses at 2;10–3;3; Garvey and Berninger, 1981). A pause of 500 ms also gives participants (especially the children) substantial time to process the lexicosyntactic and prosodic information in the utterance preceding a turn transition. The 500 ms window also allowed reliable measurement of children's anticipatory eye movements because toddlers need at least 300 ms to plan a shift in gaze (Fernald et al., 2001).

Finally, the completion part (only present in the lexicosyntactically incomplete utterances) contained between one and five words that syntactically completed the pre-silence portion. For example, “*the swings”* was extracted from “*I've seen carrots behind* ***the swings***” (Figure 1).

To avoid audibly mismatched co-articulation, we matched the place of articulation for phonemes at splicing boundaries. For example, the initial part “*I've seen*” was followed by a/k/in the recording sentence to match the initial/k/of the prosody part “*carrots behind*.” That way, when spliced together, “*I've seen*” + “*carrots behind*” had no conflicting co-articulatory cues. Similarly, we avoided co-articulatory cues to upcoming speech by controlling the phonemes immediately following incomplete prosody parts. For example, “*carrots behind*” was followed by an/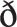/(“the”) in the recording sentence. Because the/d/in “*behind*” and the/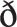/in “*the*” approximately match in place of articulation, there is no co-articulation to cue further upcoming speech. Alternately, the prosody part was followed by a phoneme with a neutral place of articulation (/Ɂ/or/h/), matched for the 500 ms silence.

We also controlled for primary stress in the two-word initial parts that had interrogative word order (*fully complete* and *incomplete prosody)*. Though the primary prosodic cue for polar interrogatives is a final high rise, they also often have high fundamental frequency at the start of the utterance (Haan, 2002). To counteract this and to also prevent the presence of prosodic boundary tones at points of intended prosodic incompleteness, we asked speakers to put emphasis on words that came late in the utterance, thereby avoiding stress placement at the start of the utterance or at the intended splicing points.

#### Stimulus pre-testing

We verified the status of our utterances as lexicosyntactically complete/incomplete with a web-based experiment using a written version of the utterances. Fourteen participants (Female = 7, mean age = 23.8 years old, native Dutch speakers) read and judged the completeness of the thirty-two (16 −SYN and 16 +SYN) target sentences up to the point of the inserted 500 ms silence (Qualtrics Software Version 55939, 2014^3^). All target sentences were found to be complete or incomplete, as intended, by more than 75% of the participants.

We verified our manipulation of prosodic completeness with a listening experiment conducted in Praat (Boersma and Weenink, 2012). Twelve participants (Female = 10, mean age = 24 years, native Dutch speakers) heard low-pass filtered versions of the target utterances (300 Hz and 50 Hz Hanning window), and were asked to judge whether each one was a question or not. Low-pass filtering removes segmental information so that only prosodic information remains. Each target utterance was presented twice, with the order of utterances fully randomized. Eleven (34%; five complete and six incomplete) targets were judged differently than intended (e.g., judged as an interrogative contour when it should have been non-interrogative, or vice versa) in more that 25% of the judgments. These ambiguous prosodic contours were therefore taken into account during data analysis and interpretation.

#### Video stimuli

Two pairs of puppets were used to create the stimulus videos. To match the puppet videos to the audio stimuli as closely as possible, two puppeteers listened to the dialogs and simultaneously moved the puppet mouths during video recording. The puppeteers aimed to complete an open-close mouth movement for each syllable in the recording. With the exception of mouth movements, the puppets were immobile. We then combined the puppet video recordings with the audio stimuli, maximizing the quality of sound and speech alignment in Adobe Premiere Pro video editing software.

#### Data pre-processing

Before analyzing children's anticipatory gaze switches, the raw data set was pre-processed to remove unreliable tracker output and to prepare gaze measurements for the main gaze-switch analysis. We only counted participants' gaze measurements when the Tobii output marked the look as valid in at least one eye. Trials were excluded when a participant attended to the screen for less than 75% of the total trial duration. If this happened for more than four trials, the participant's data was completely excluded from further analysis because of a general inattention to the stimuli. In total, eleven toddlers (33%) were completely excluded by this criterion. No adults were completely excluded. From the remaining participants (21 toddlers and 16 adults), 27 trials (2.5%) were excluded in total (toddlers: 21, adults: 6). The final dataset contained gaze data for 1056 trials.

Our main question was how toddlers use linguistic cues in their *prediction* of upcoming speaker changes, so we only analyzed gaze switches that were initiated before children could have reacted to speaker continuation/speaker switch. We used an algorithm for switch identification developed by Casillas and Frank (under review). According to this three-step checklist, switches are anticipatory if they fulfill the following criteria: (1) a participant fixates on the prior speaker for at least 100 ms at the end of the prior turn, (2) sometime thereafter the participant switches to fixate on the upcoming speaker for at least 100 ms and, (3) a gaze shift is initiated within the first 300 ms of the response turn for toddlers (Fernald et al., 2001), or 200 ms for adults^4^.

**Figure 2:**
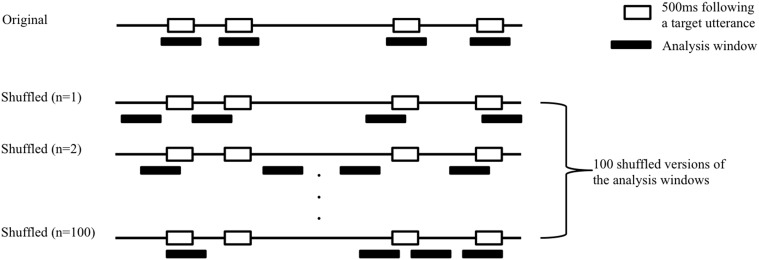
**Shuffling procedure for estimating random baseline anticipatory switches**. We created 100 randomly shuffled versions of the original analysis window placement. The horizontal line represents the duration of one example dialog. The white boxes on the line represent the four 500 ms silences after potential target turn transitions (32 in total across the 8 dialogs; See Table 1). The black boxes beneath the line represent the four analysis windows for each target 500 ms silence. In the original version, the analysis windows are centered on the target 500 ms silences. In the shuffled versions, the analysis windows are randomly redistributed over the duration of the turn. We then re-ran the gaze identification algorithm, pairing the eye tracker data with each randomly shuffled version to estimate the baseline probability of making an anticipatory shift when the analysis windows are uncoupled from the actual target 500 ms silences.

**Figure 3:**
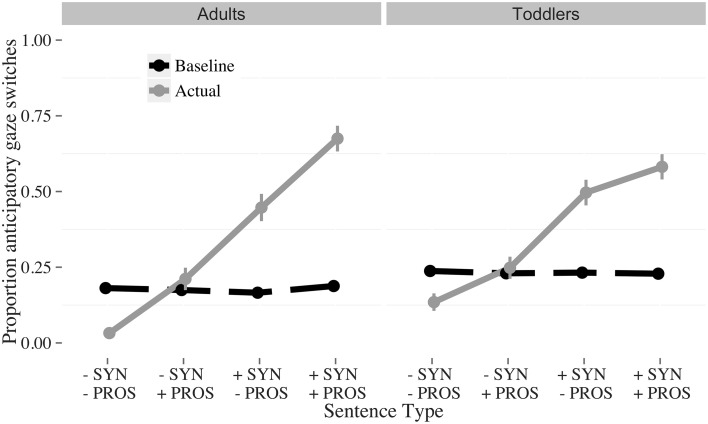
**Proportion of baseline (dashed) and actual anticipatory (solid) gaze switches to the answerer, by condition and age**. The conditions were: *Fully incomplete* (−SYN −PROS); *Incomplete syntax* (−SYN +PROS); *Incomplete prosody* (+SYN −PROS); *Fully complete* (+SYN +PROS). The vertical bars indicate the standard error of the mean.

Random gaze switches between speakers can sometimes, by chance, conform to these three criteria, and could therefore be mistakenly categorized as “true” gaze switches. Therefore, we estimated and corrected for participants' baseline random anticipatory looking behavior. Again, algorithmic details were borrowed from Casillas and Frank (under review). We ran each participant's actual eye-tracking data through the exact same switch identification algorithm (described above), but this time with 100 randomly-shuffled versions of the original turn-transitions in the videos (Figure 2). The idea was that, if we assume as our null hypothesis that children's switching behavior is random, their rates of anticipatory switching should be the same no matter where we place our analysis windows (at real turn transitions vs. anywhere else in the stimulus). We therefore made 100 versions of the original analysis windows in which the original analysis windows for each stimulus were distributed randomly between its start and end time (Figure 2). Then, using the three-step algorithm described above, we determined whether the participant made an anticipatory switch or not for each turn transition in each randomly-shuffled version. This procedure was repeated 100 times. Then we averaged the results to get a single baseline estimate of random switching for each target turn transition for each participant. We then obtained corrected anticipatory gaze switch values by subtracting the random anticipatory gaze switch value from the original gaze switch value for each turn transition for each participant. These corrected anticipatory switch values were then used in all statistical analyses (see also Figures 3, **5**).

### Results

The complete pre-processed dataset (toddlers and adults together) was analyzed using linear mixed effect models (lme4; Bates et al., 2012) in the statistical programming language R (R Development Core Team, 2013). Significance of the predictors was evaluated by using the obtained *z*-score as a *t*-statistic (|*t*| > 1.96 is significant at α = 0.05).

#### Pre-analysis: random anticipatory looking

The original anticipatory gaze switches and the random baseline anticipatory gaze switches are visualized in Figure 3. Participants switch less than would be expected by chance in the *fully incomplete* (−SYN, −PROS) condition, at chance level for the *incomplete syntax* (−SYN, +PROS) condition, and above chance for both the *incomplete prosody* (+SYN, −PROS) and *fully complete* (+SYN, +PROS) conditions. This pattern suggests that participants use both lexicosyntactic and prosodic cues for turn-projection: When both cues are incomplete, participants do not expect a speaker change, whereas when both cues are complete, they do. When the cues are pitted against each other, listeners weigh lexicosyntactic over prosodic cues.

#### Lexicosyntactic and prosodic cues

In order to assess the effects of linguistic cue and participant age, we first fit a model to participants' baseline-corrected anticipatory switches (1056 observations; *N* = 37; Table 2). All targets (*N* = 32) were included in the model. Recall that the prosodic pre-test (Section Conversation Design) showed that eleven targets had ambiguous prosodic contours. A model including these ambiguous targets did not reveal qualitatively different results compared to a model excluding these targets. Therefore, the final model included all targets. The dependent variable was participants' baseline-corrected anticipatory gaze switches. Predictor variables included *syntactic completeness* (incomplete vs. complete), *prosodic completeness* (incomplete vs. complete) and *age* (toddler vs. adult). The predictor variables were contrast-coded (Table 2) and the intercept was allowed to vary by subject and item.

**Table 2:** **Outcomes from the linear mixed effects model including both subject groups (Dutch toddlers and adults; Number of observations: 1056; *N* = 37)**.

**Predictor**	**Contrast coding**	*β*	***t (z)***	***p***
Intercept		0.15	4.77	
Syntactic completeness	Incomplete (−1) Complete (1)	0.20	6.67	<0.001
Prosodic completeness	Incomplete (−1) Complete (1)	0.075	2.56	<0.05
Age	Toddler (−1) Adult (1)	0.019	1.28	
Syntactic completeness × Prosodic completeness		−0.0015	−0.051	
Syntactic completeness × Age		0.024	2.03	0.05
Prosodic completeness × Age		0.022	1.82	
Syntactic completeness × Prosodic completeness × Age		0.0065	0.54	

The amount of linguistic information consistent with turn completion affected participants' anticipatory switching. Model coefficients show three significant effects in the anticipatory gaze data. First, the proportion of anticipatory gaze switches was larger for the lexicosyntactically complete vs. incomplete targets (β = 0.20, *z* = 6.67, *p* < 0.001). Second, more anticipatory gaze switches were made for complete prosodic contours than for incomplete prosodic contours (β = 0.075, *z* = 2.56, *p* < 0.05). Third, there was an interaction between *syntactic completeness* and *age* (β = 0.024, *z* = 2.03, *p* = 0.05). No other coefficients reached significance.

**Figure 4:**
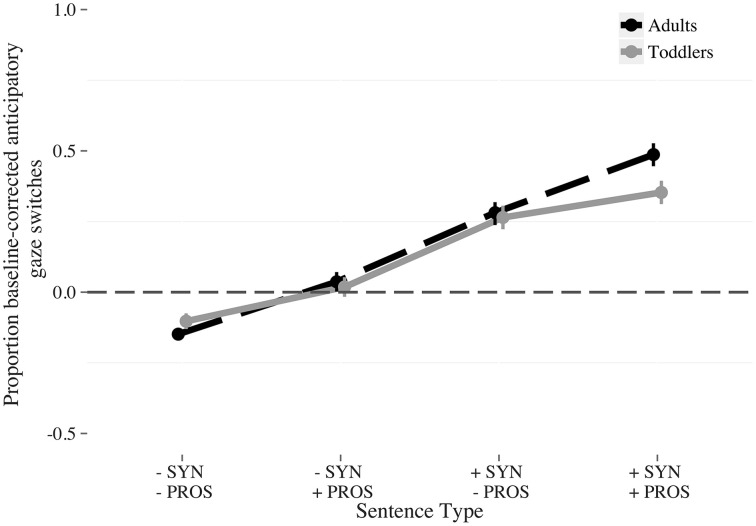
**Proportion baseline-corrected anticipatory switches to the answerer by condition and age (Adults: dashed; Toddlers: solid)**. The conditions were: *Fully incomplete* (−SYN −PROS); *Incomplete syntax* (−SYN +PROS); *Incomplete prosody* (+SYN −PROS); *Fully complete* (+SYN +PROS). The vertical bars indicate the standard error of the mean.

Visual inspection of the data (Figure 4) suggests that interaction between syntactic completeness and age comes from the *fully complete* condition, in which toddlers and adults differed in their overall number of anticipatory switches (adults switch more than toddlers do). We fitted a model restricted to the syntactically complete conditions (*fully complete* and *incomplete prosody*, 528 observations, *N* = 37, Table 3) to test this hypothesis. No significant effect of *age* was found. Therefore, this explanation for the interaction was not verified in the statistical model.

**Table 3:** **Result from the linear mixed effects models for Dutch toddlers and adults by lexicosyntactic complete targets (528 observations, *N* = 37)**.

**Predictor**	**Contrast coding**	*β*	***t(z)***	***p***
Intercept		0.34	5.71	
Prosodic completeness	Incomplete (−1) Complete (1)	0.074	1.29	
Age	Toddler (−1) Adult (1)	0.044	1.70	
Age × Prosodic completeness		0.028	1.52	

#### Relative weight of lexicosyntactic and prosodic cues

A second model was fit to test the relative weight of lexicosyntactic and prosodic cues. We restricted the data to the two partially complete conditions (*incomplete syntax* and *incomplete prosody*). The predictor variables were *condition* (incomplete syntax vs. incomplete prosody) and *age* (toddler vs. adult). Again, the intercept was allowed to vary by subject and item.

The model showed that participants weighed lexicosyntactic cues over prosodic cues. Participants made more anticipatory gaze switches after utterances with complete syntax (*incomplete prosody)* compared to utterances with complete prosody (*incomplete syntax*; β = 0.12, *z* = 2.62, *p* < 0.05; Table 4). No other predictors reached significance.

**Table 4:** **Outcomes from the linear mixed effects model of the two partially complete conditions for both subject groups (*incomplete syntax*, *incomplete prosody*, Number of observations: 528; *N* = 37)**.

**Predictor**	**Contrast coding**	β	***t (z)***	***p***
Intercept		0.15	3.14	
Condition	Incomplete syntax (−1) Incomplete prosody (1)	0.12	2.62	<0.05
Age	Toddler (−1) Adult (1)	0.012	0.57	
Condition × Age		0.0026	0.15	

#### Speaker change or speaker continuation

Recall that lexicosyntactically complete targets were followed by a change in speakership 50% of the time. Ideally there should be no difference between cases of speaker change and speaker continuation. If there were a difference, it could indicate that participants' gaze switches were triggered by the mouth movement of the responding or continuing puppet rather than by the participants' predictions alone.

We ran an additional analysis to test whether anticipatory gaze switches were influenced by speaker change in the lexicosyntactically complete conditions (*fully complete* and *incomplete prosody*), with speaker continuation (-1) and speaker change (1) contrast-coded. The analysis was restricted to the lexicosyntactically complete conditions since syntactically incomplete targets were always followed by continuation of the same speaker.

The model (528 observations, *N* = 37) revealed a significant effect of *speaker change*. Participants made more anticipatory gaze switches when the target was followed by a speaker change compared to a speaker continuation (β = 0.18, *z* = 5.26, *p* < 0.001; Table 5). No other coefficients reached significance.

**Table 5:** **Results from the linear mixed effect models for Dutch toddlers and adults in the lexicosyntactically complete conditions, including the predictor variable speaker change (528 observations, *N* = 37)**.

**Predictor**	**Contrast coding**	β	***t (z)***	***p***
Intercept		0.34	8.88	
Prosodic completeness	Incomplete (−1) Complete (1)	0.074	2.16	<0.05
Age	Toddler (−1) Adult (1)	0.044	1.69	
Speaker change	No (−1) Yes (1)	0.18	5.26	<0.001
Prosodic completeness × Age		0.029	1.54	
Prosodic completeness × Speaker change		−0.010	−0.31	
Age × Speaker changes		0.022	1.17	
Prosodic completeness × Age × Speaker change		−0.0078	−0.42	

A closer look at the video stimuli indeed showed that sound and mouth movement were not adequately aligned in almost half of the syntactically complete target utterances: In nine of the sixteen lexicosyntactically complete target utterances, mouth movement preceded the onset of the audio signal by more than a few milliseconds. This early mouth movement could have triggered participants' gaze shifts toward the moving puppet, regardless of the linguistic content available.

Additionally, because the prior speaker *always* continued after the silence for lexicosyntactically incomplete targets, but only continued 50% of the time after lexicosyntactically complete targets, there was a statistical bias in the stimuli that could have caused participants to make fewer anticipatory gaze switches for lexicosyntactically incomplete targets. If so, participants would have to learn this statistical bias during the course of the experiment; it should only be present at the end of the experiment. We fit two linear mixed effect models to (a) data from the first two trials (268 observations, *N* = 36) and (b) data from the last two trials (248 observations, *N* = 34). In both models, the main effect of lexicosyntactic completeness was present (First two trials: β = 0.146; *z* = 3.052, *p* < 0.01; Last two trials: β = 0.199, *z* = 4.885, *p* < 0.0001, Table 6). The results therefore do not support statistical learning as a basis for the effects of lexicosyntactic completeness.

**Table 6:** **Outcomes from the main linear mixed effects model for (A): first two trials of the experiment and (B): last two trials of the experiment (Dutch toddlers and adults)**.

	**Dutch**					
	**(A). First two trials**			**(B). Last two trials**		
	**268 observations, *N* = 36**			**248 observations, *N* = 33**		
	β	***t (z)***	***p***	β	***t (z)***	***p***
Intercept	0.114	2.364		0.0984	2.225	
Syntactic completeness	0.146	3.052	<0.01	0.199	4.885	<0.0001
Prosodic completeness	0.0382	0.798		0.0847	2.080	<0.05
Age	0.0301	1.130		0.000563	0.020	
Syntactic completeness × Prosodic completeness	−0.701	−1.465		0.0242	0.595	
Syntactic completeness × Age	0.0224	0.866		−0.0131	−0.575	
Prosodic completeness × Age	0.0192	0.744		0.0115	0.503	
Syntactic completeness × Prosodic completeness × Age	−0.679			0.0335	1.470	

### Discussion

Both Dutch toddlers and adults used lexicosyntactic and prosodic cues in their anticipation of upcoming speaker changes. Participants made the least anticipatory gaze switches when both cues signaled an incomplete turn. The most anticipatory gaze switches were made when both cues signaled a complete turn. When the lexicosyntactic and prosodic cues were pitted against each other (*incomplete syntax* and *incomplete prosody*), listeners weighed lexicosyntactic over prosodic cues.

The advantage for lexicosyntactic over prosodic cues in turn-projection is consistent with prior work on adult turn-taking (Caspers, 2001; de Ruiter et al., 2006), but was unexpected for toddlers. Recent work on children's use of prosodic and lexicosyntactic cues in predicting upcoming turn structure found an early global advantage for prosodic over lexicosyntactic cues in 1- and 2-year-olds (Casillas and Frank, 2013). An early advantage for prosodic cues would have also been consistent with the general pattern in language acquisition that sensitivity to prosodic cues precedes sensitivity to lexicosyntactic cues (Nazzi et al., 1998; Christophe et al., 2008).

Before accepting the hypothesis that 2.5-year-old toddlers weigh lexicosyntactic over prosodic cues in their turn-projection, alternative explanations need to be explored. A first explanation relates to the reliability of the prosodic contours in the stimuli. Recall that 11 of the 32 prosodic contours were ambiguous in whether they signaled interrogativity (completeness) or not; pre-test participants classified these 11 contours incorrectly at least 25% of the time. The results of the main experiment did not qualitatively shift when these ambiguous prosodic contours were included (Section Lexicosyntactic and Prosodic Cues), but their presence could have affected overall task performance. For example, toddlers may have noticed that the prosodic contours were strange or unclear and therefore unconsciously shifted their attention away from the prosodic cues in favor of the (less ambiguous) lexicosyntactic cues.

Another alternative explanation for toddlers' reliance on lexicosyntactic cues is that the puppet movements gave unintentional cues to turn hold or turn transition. *Post-hoc* analyses revealed that participants made more anticipatory gaze switches when lexicosyntactically complete turns were followed by a change in speakership compared to when they were followed by a continuation of the same speaker. We found that non-verbal cues (e.g., opening mouth, movements) preceded the onset of the acoustic signal in 9 of the 16 syntactically complete target utterances. These early non-verbal cues could have enhanced the effect of lexicosyntactic completeness, because early visual cues to speaker change were available in some of the lexicosyntactically complete target utterances, while lexicosyntactically incomplete target utterances were never followed with visual cues to speaker change (the same speaker always continued; Table 1).

Despite these methodological issues, the results from Experiment 1 still suggest that lexicosyntactic cues are weighed over prosodic ones in children's prediction of upcoming turn structure. To test the robustness (non-language specificity) of these findings, we conducted a second experiment with British English toddlers and adults.

## Experiment 2

Experiment 2 tested how English-speaking toddlers weigh prosodic and lexicosyntactic cues for upcoming turn structure prediction. Diverging slightly from Experiment 1, the recording and splicing for the target utterances in Experiment 2 used an extra criterion: the “prosody part” of the target utterances contained at least four syllables (only two were used in Experiment 1; see Section Stimulus Preparation). By extending the prosodic contour over more syllables, we gave the listener more time to perceive the contour being used. We derived the criterion of “four syllables” from the Dutch pre-test for prosodic completeness; most of the errors were made on *prosody parts* with fewer than four syllables. As in Experiment 1, participants' eye movements were recorded as they watched eight videos of dyadic puppet conversation.

### Materials and methods

#### Participants

Twenty-five native British English-speaking 2.5-year-olds participated in the experiment. Of these, five were excluded because of equipment error (1) and inattention to the screen during the experiment (4; see Section Data Pre-Processing). As a result, 20 toddlers were included in the final set for analysis (Female = 10, mean age = 29 months, range = 25–33 months). Twenty adult participants (native British English-speakers, Female = 13, mean age = 21 years) participated as a control group. No participants reported hearing or vision problems. Ethical approval was obtained via the Ethics Committee for the School of Humanities and Social Sciences of the University of Cambridge.

#### Apparatus and procedure

All participants were tested in the Psycholinguistics Lab of the Department of Theoretical and Applied Linguistics in Cambridge, UK. Eye-tracker calibration and stimulus presentation were controlled by Tobii Studio 3.2.1.190 software. The data were obtained with a Tobii X120 infrared eye-tracking camera (Tobii Technology AB; binocular infrared light reflection, 120 Hz sampling frequency, accuracy range: 0.5° to 1°, recovery <300 ms). The camera was placed below a 17-inch monitor and calibrated for distance and angle relative to the monitor. The experimental procedure was the same as for Experiment 1.

#### Materials

#### Target sentences

Target sentences were created and spliced using the same procedure as in Experiment 1 (Section Audio Stimuli; Table 1). Again, we verified the lexicosyntactic completeness of the targets with a web-based experiment of the sentences in written form (*N* = 14, female = 8, mean age = 29 years old, native British English speakers). All targets were found to be complete or incomplete, as intended, by more than 75% of the participants. Also as before, we conducted a prosodic completeness listening pre-test (Praat; Boersma and Weenink, 2012; *N* = 12, female = 10, mean age = 24 years, native British English speakers), which showed that only two (both prosodically complete) target sentences had ambiguous prosody. Their contours were judged as non-interrogative instead of interrogative in more that 25% of the judgments.

#### Conversation and video construction

Conversations in Experiment 2 (English) were not restricted to word-for-word translations of the conversations in Experiment 1 (Dutch) to allow for more freedom in using child-friendly and culturally appropriate topics (Fenson et al., 1993). However, the structure (turns and placement of conditions) and length (30s) of the conversations were identical between the two experiments.

Audio recordings were obtained using the same procedure from Experiment 1, but with two female native Southern British English speakers (the local dialect in the testing region).

The same pairs of puppets were used from Experiment 1. As before, we created puppet video recordings to match the final audio stimuli. All video recordings were edited for speech alignment and sound quality in Adobe Premiere Elements video editing software. If perfect alignment of sound and movement could not be achieved, the audio signal always preceded the video movement, so that movement of the mouths could not be used as an anticipatory cue for turn transition. This criterion was added to avoid an effect of visual cues to turn transition on participants' looking behavior.

#### Data pre-processing and analysis

The same criteria and algorithms were used as in Experiment 1 for participant exclusion, anticipatory gaze switche identification, and random-baseline correction of switching values (Section Data Pre-Processing; Figure 2). In total, four toddlers (16%) were completely excluded from the analyses for inattention to the screen. No adults were completely excluded. Of the remaining participants (20 toddlers, 20 adults), 27 trials (2.3%) were excluded (25 for the toddlers and two for the adults). The final data set contained gaze data for 1144 trials.

### Results

#### Pre-analysis: random anticipatory looking

**Figure 5:**
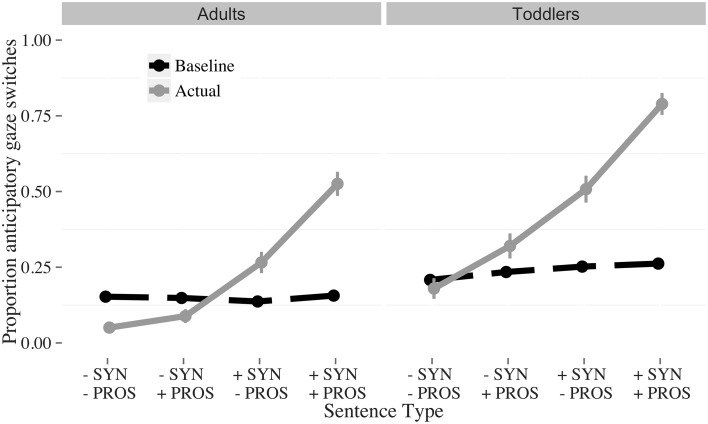
**Proportion of baseline (dashed) and actual anticipatory (solid) gaze switches to the answerer, by condition and age**. The conditions were: *Fully incomplete* (−SYN −PROS); *Incomplete syntax* (−SYN +PROS); *Incomplete prosody* (+SYN −PROS); *Fully complete* (+SYN +PROS). The vertical bars indicate the standard error of the mean.

Participants switched less than would be expected by chance in the *fully incomplete* (-SYN -PROS) condition, at chance level for the *incomplete syntax* (−SYN +PROS) condition, and above chance for both the *incomplete prosody* (−SYN +PROS) and *fully complete* (+SYN +PROS) conditions (Figure 5). This pattern again suggests that participants use both lexicosyntactic and prosodic cues for turn-projection. As in Experiment 1, when both cues were incomplete, participants were least likely to expect a speaker change, whereas when both cues were complete, they were the most likely to expect one. When the cues were pitted against each other, listeners weighed lexicosyntactic over prosodic cues.

#### Lexicosyntactic and prosodic cues

A model similar to that fitted in Experiment 1 assessed the effects of linguistic cues and age on participants' baseline-corrected anticipatory gaze switches (1144 observations, *N* = 40; Table 7). The dependent variable was participants' baseline-corrected anticipatory switches. Predictor variables included *syntactic completeness* (incomplete vs. complete), *prosodic completeness* (incomplete vs. complete) and *age* (toddler vs. adult). The intercept was allowed to vary by subject and item, and the predictor variables were contrast-coded (−1, 1).

**Table 7:** **Outcomes from main linear mixed effects model for both subject groups (English toddlers and adults; Number of observations: 1144, *N* = 40)**.

**Predictor**	**Contrast coding**	β	***t (z)***	***p***
Intercept		0.15	7.88	
Syntactic completeness	Incomplete (−1) Complete (1)	0.17	10.55	<0.001
Prosodic completeness	Incomplete (−1) Complete (1)	0.084	5.12	<0.001
Age	Toddler (-1) Adult (1)	−0.062	−4.37	<0.001
Syntactic completeness × Prosodic completeness		0.044	2.71	<0.05
Syntactic completeness × Age		−0.0084	−0.74	
Prosodic completeness × Age		−0.013	−1.34	

**Figure 6:**
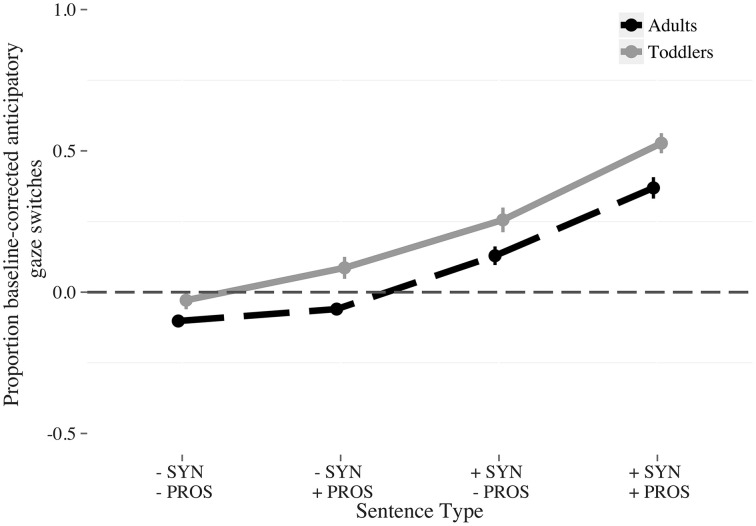
**Proportion baseline-corrected anticipatory switches to the answerer by condition and age (Adults: dashed; Toddlers: solid)**. The conditions were: *Fully incomplete* (−SYN −PROS); *Incomplete syntax* (−SYN +PROS); *Incomplete prosody* (+SYN −PROS); *Fully complete* (+SYN +PROS). The vertical bars indicate the standard error of the mean.

Again we found that the amount of linguistic information consistent with turn completion affected participants' anticipatory switching (Figure 6). Model coefficients show four significant effects (Figure 6; Table 7). First, the proportion of anticipatory gaze switches was larger for the lexicosyntactically complete vs. lexicosyntactically incomplete targets (β = 0.17, *z* = 10.55, *p* < 0.001). Second, more anticipatory gaze switches were made for complete prosodic contours vs. incomplete prosodic contours (β = 0.084, *z* = 5.12, *p* < 0.001). Third, toddlers made more anticipatory gaze switches overall than adults (β = −0.062, *z* = −4.37, *p* < 0.001). Fourth, there was an interaction between syntactic completeness and prosodic completeness (β = 0.044, *z* = 2.71, *p* < 0.05). No other coefficients reached significance.

We fit separate *post-hoc* models of the lexicosyntactically complete target sentences (*fully complete* and *incomplete prosody)* and the lexicosyntactically incomplete target sentences (*fully incomplete* and *incomplete syntax*) to explain the interaction between syntactic completeness and prosodic completeness. A model restricted to syntactically complete target sentences (572 observations, *N* = 40; Table 8) showed a significant effect of *prosodic completeness*, with more anticipatory gaze switches for prosodically complete than prosodically incomplete contours (β = 0.13, *z* = 4.92, *p* < 0.001). In comparison, a model restricted to syntactically incomplete targets (572 observations, *N* = 40; Table 8), only showed a marginal effect of prosodic completeness (β = 0.040, *z* = 1.99, *p* = 0.05). These *post-hoc* analyses reveal that English listeners' use of prosodic cues depends on whether the utterances are syntactically complete; when utterances were lexicosyntactically incomplete, the effect of prosody was only marginally significant.

**Table 8:** **Results from the linear mixed effect models for toddlers and adults together by lexicosyntactic condition (complete or incomplete, number of observations for each condition: 572, *N* = 40)**.

**Lexicosyntactic condition**	**Complete**			**Incomplete**		
**Predictor**	β	***t(z)***	***p***	β	***t(z)***	***p***
Intercept	0.32	11.07		−0.027	−1.30	
Prosodic completeness	0.13	4.92	<0.001	0.040	1.99	0.05
Age	−0.071	−3.22	<0.01	−0.054	−3.61	<0.001
Age × Prosodic completeness	−0.008	−0.44		0.018	−1.33	

#### Relative weight of lexicosyntactic and prosodic cues

Similar to Experiment 1, we then fit a second model restricted to the two partially complete conditions (*incomplete syntax* and *incomplete prosody*) to test the relative weight of lexicosyntactic and prosodic cues (484 observations, *N* = 40; Table 9). The predictor variables were *condition* (incomplete syntax vs. incomplete prosody) and *age* (toddler vs. adult).

**Table 9:** **Outcomes from the linear mixed effects model for the two partially complete conditions for both subject groups (incomplete prosody, incomplete syntax, Number of observations: 572, *N* = 40)**.

**Predictor**	**Contrast coding**	β	***t (z)***	***p***
Intercept		0.10	3.63	
Condition	Incomplete syntax (−1) Incomplete prosody (1)	0.089	3.29	<0.01
Age	Toddler (−1) Adult (1)	−0.067	−3.67	<0.001
Condition × Age		0.0045	0.27	

The model showed that participants weighed lexicosyntactic cues over prosodic cues; they made more anticipatory gaze switches when they only had complete syntax (*incomplete prosody*) compared to when they only had complete prosody (*incomplete syntax*; β = 0.89; *z* = 3.29, *p* < 0.01).

#### Speaker change or speaker continuation

We fit an additional model to the baseline-corrected switches in lexicosyntactically complete target utterances (572 observations, *N* = 40; Table 10) in order to check whether more anticipatory gaze switches were made when there was a change in speakership compared to when there was no change in speakership. The model suggested no effect of speaker change/continuation (β = 0.039, *z* = 1.53, *p* = n.s) Therefore, the effect of lexicosyntax in Experiment 2 cannot be attributed to early visual cues of speaker change (as was possible in Experiment 1).

**Table 10:** **Results from the linear mixed effect models for English toddlers and adults together in the lexicosyntactically complete conditions, including the predictor variable speaker change (Number of observations: 572, *N* = 40)**.

**Predictor**	**Contrast coding**	β	***t (z)***	***p***
Intercept		0.32	11.23	
Prosodic completeness	Incomplete (−1) Complete (1)	0.13	4.99	<0.001
Age	Toddler (−1) Adult (1)	−0.071	−3.21	<0.01
Speaker change	No (−1) Yes (1)	0.039	1.53	
Prosodic completeness × Age		−0.0076	−0.41	
Prosodic completeness × Speaker change		0.018	−0.70	
Age × Speaker changes		−0.018	−1.01	
Prosodic completeness × Age × Speaker change		−0.014	−0.74	

Finally, we fit two models to data from (a) the first two trials (292 observations, *N* = 38) and (b) the last two trials (252 observations, *N* = 33) of the experiment to test whether children learned to not switch after lexicosyntactically incomplete utterances during the course of the experiment. As in Experiment 1, the *post-hoc* tests showed significant main effects of lexicosyntactic completeness in both the first two trials (β = 0.174, *z* = 4.574, *p* < 0.0001) and the last two trials (β = 0.183, *z* = 7.252, *p* < 0.0001, Table 11). It is therefore unlikely that our findings for lexicosyntactic completeness were driven by a learned statistical bias.

**Table 11:** **Outcomes from the main linear mixed effects model for (A): first two trials of the experiment and (B): last two trials of the experiment (English toddlers and adults)**.

	**English**					
	**(A). First two trials 292 observations, *N* = 38**			**(B). Last two trials 252 observations, *N* = 38**		
	***β***	***t (z)***	***p***	***β***	***t (z)***	***p***
Intercept	0.134	0.0381		0.106	3.973	
Syntactic completeness	0.174	4.574	<0.0001	0.183	7.252	<0.0001
Prosodic completeness	0.0833	2.187	<0.05	0.068	3.827	<0.001
Age	−0.0728	−3.233	<0.01	−0.0351	−1/394	
Syntactic completeness × Prosodic completeness	0.0304	0.797		0.0486	1.920	
Syntactic completeness × Age	0.00680	0.302		−0.0176	0.742	
Prosodic completeness × Age	−0.0114	−0.505		−0.0146	−0.619	
Syntactic completeness × Prosodic completeness × Age	0.0167	0.755		−0.0146	−0.619	

### Discussion

The second experiment showed a very similar pattern of findings to Experiment 1: Toddlers and adults used both lexicosyntactic and prosodic cues for turn projection. Also, both English toddlers and adults weighed lexicosyntactic cues over prosodic cues when the two were pitted against each other.

One difference in the results from Experiments 1 and 2 is that, in Experiment 2 (English), toddlers made more anticipatory gaze switches than adults. This effect of children switching more often than adults has been previously observed in studies with a similar design (Casillas and Frank, 2012), having been explained as an effect of the videos being easy to follow. The explanation is that adults find the videos easy to comprehend and therefore track the turn structure less closely with their eye movements. This explanation fits with our findings in that the presence of ambiguous prosodic contours in Experiment 1 may have made the task more difficult for Dutch adults, leading them to track the conversations more closely than adults did in the clearer, easier contours in the English stimuli.

A second difference between Experiments 1 and 2 is that English listeners used prosodic cues when utterances were lexicosyntactically complete, but not when they were incomplete. This effect was not observed for the Dutch listeners, but it is consistent with prior experimental work on English (Wichmann and Caspers, 2001).

## General discussion

In two experiments, we investigated toddlers' and adults' use of lexicosyntactic and prosodic cues in making predictions about upcoming turn structure. The experiments were conducted in two languages, Dutch and British English, to test whether the findings were based on language-specific cues for turn prediction. Adults and toddlers in both languages used both lexicosyntactic and prosodic cues in their anticipation of upcoming speaker changes. Participants made the most anticipatory gaze switches when both cues were complete and interrogative (*fully complete*). Participants also anticipated upcoming speaker changes when the lexicosyntactic cue alone was complete and interrogative (*incomplete prosody*). Importantly, complete lexicosyntax alone was not equivalent to the combined effect of complete lexicosyntax and prosody; participants showed a benefit for prosody in that the *fully complete* targets elicited more anticipatory gaze switches than the targets with complete lexicosyntax alone (*incomplete prosody*).

When only the prosodic cue was complete and interrogative (*incomplete syntax*), participants' anticipatory gaze switches did not differ from chance. Participants made the fewest anticipatory gaze switches when both cues were incomplete and declarative (*fully incomplete*), making fewer gaze switches than would be expected by chance. This last finding is the first to demonstrate that toddlers know when *not* to switch; they keep their eyes on the current speaker more often when lexicosyntactic and prosodic cues both signal an incomplete turn.

Our general finding, that listeners use both lexicosyntactic and prosodic cues for turn-projection (but weigh lexicosyntactic information above prosody overall), is compatible with previous findings showing an advantage for *combined* lexical and prosodic cues over lexical cues alone (Duncan, 1972; Ford and Thompson, 1996; Casillas and Frank, 2012, 2013).

### Lexicosyntactic vs. prosodic cues

We tested the relative weight of lexicosyntactic and prosodic cues by pitting them directly against each other in two conditions (*incomplete syntax* and *incomplete prosody*). Adults were expected to privilege lexicosyntactic information above all (de Ruiter et al., 2006; Magyari and de Ruiter, 2012), while toddlers were expected to privilege prosodic cues instead (Gleitman and Wanner, 1982; Morgan and Demuth, 1996; Jusczyk, 1997; Christophe et al., 2008; Casillas and Frank, 2013). Contrary to our expectations, adults and toddlers did not differ in their relative cue weights; both showed a privilege for lexicosyntactic over prosodic cues in their predictions. There are at least four reasons why this finding could have arisen, three derive from the design of our study and one from the use of prosody for other functions.

Participant's expectations about upcoming turn structure were maximally contrasted for our *fully incomplete* (no speaker switch expected) and *fully complete* (speaker switch expected) conditions. Targets in the *fully incomplete* condition were always declaratives whereas targets in the *fully complete* condition were interrogatives. Interrogatives automatically cue a speaker switch whereas declaratives don't. As a result, it is important to keep in mind that (by design) the stimuli confounded completeness with interrogative status: both the lexicosyntactic and the prosodic cues to completeness created interrogative utterances, whereas the cues to incompleteness created declarative utterances. Previous work suggests that infants are already sensitive to lexicosyntactic and prosodic cues to questionhood by age two, and that they treat interrogatives differently from declaratives (Lexicosyntax: Shi et al., 2006; Casillas and Frank, 2012, 2013; Geffen and Mintz, 2014; Prosody: Soderstrom et al., 2011; Combined cues: Casillas and Frank, 2012, 2013; Geffen and Mintz, 2012). This pattern continues through adulthood; adults in conversation also give special attention to questions (or other acts eliciting a response; Stivers and Rossano, 2010). In our study, the lexicosyntactic cues to questionhood (subject-auxiliary inversion and *do*-insertion in English) appeared earlier in the utterance than the prosodic cue to questionhood (final high rise). Therefore, it is possible that children weigh lexicosyntactic cues over prosodic cues simply because the lexicosyntactic cues appear earlier than the prosodic ones in the utterances—not because they find lexicosyntactic cues more informative or more important overall. Because our lexicosyntactically complete targets were always formatted as questions (whereas our lexicosyntactically incomplete targets were always formatted as declaratives), the main effect of lexicosyntax could therefore have been driven by a higher response pressure for questions vs. declaratives, instead of for lexicosyntactic complete vs. incompleteness. The current results leave *which* lexicosyntactic cues toddlers used for prediction—completeness, interrogativity, or a combination of the two—as an open question for future research.

As they stand, the current results add to the evidence that toddlers not only distinguish between interrogative and declarative word order (Geffen and Mintz, 2014), but that they are also sensitive to the difference in function between declarative and interrogative utterances. As seen in similar work (Casillas and Frank, 2012, 2013) toddlers made more anticipatory gaze switches after interrogatives compared to declaratives, suggesting that they expect the addressee to reply when a question is (lexicosyntactically) introduced.

A second explanation for toddlers' use of lexicosyntactic cues over prosodic ones is that the lexicosyntactic cues to turn completeness were more consistent in their interpretation (and therefore more reliable) compared to the prosodic cues to turn completeness. Although we took care to select prosodic cues that are relatively consistent and prototypical in signaling a speaker switch (high rising terminal contours to signal interrogativity), rising pitch at prosodic boundaries can, in principle, signal multiple different meanings. There is no one-to-one mapping between intonational contours and their pragmatic function in conversational contexts. Thus, the form-function mappings for prosodic cues may have been less straightforward compared to the mappings for lexicosyntactic cues.

A third, related, explanation derives from a difference in the pragmatic felicity of the two partially complete conditions (*incomplete prosody* and *incomplete syntax*): the *incomplete prosody* condition is less marked than the *incomplete syntax* condition. In natural conversation, it is common for lexicosyntactically complete phases to lack prosodic boundaries (e.g., when the syntactic phrase optionally continues beyond the first possible completion point). But questioning contours rarely occur when lexicosyntax is incomplete, unless they are specifically conditioned by contexts where (a) the addressee is making a repair (Did you mean to say, *“That's a very high”?*) or (b) the speaker is trying to elicit a sentence completion from the addressee, as parents often do with young children during word-elicitation games (e.g., *“A pig says ‘oink’ and a cow says?”*) As a consequence, it might have been more difficult to understand the *incomplete syntax* target sentences compared to the *incomplete prosody* sentences, thereby explaining the fewer anticipatory gaze switches in the *incomplete syntax* condition without any reference to cue dominance.

A fourth explanation is that prosodic and lexicosyntactic cues are used differently to signal linguistic function, either from the point of view of the *type* of linguistic information being conveyed, or the *extent* of its predictive domain in conversational interaction. Although many studies have shown that children are capable of perceptually distinguishing the types of intonational contours used in the current study (even before the acquisition of segmental and syntactic structure; Snow and Balog, 2002), it is unclear how much of the prosodic system can be acquired before children also master other aspects of the linguistic system. The acquisition of an intonational system involves much more than the ability to produce and discriminate rising and falling pitch movements. Children must also be able to map pitch contours to functional meanings. This involves learning the language's inventory of phonologically distinct intonational contours (e.g., rising, falling, rising-falling, etc…), figuring out what their linguistic and paralinguistic functions are (e.g., rising for interrogativity, but also continuation, etc…), determining how they are realized within utterances (e.g., throughout a phrase, or only in the accented syllable), and finding out what determines variation in their phonetic implementation (e.g., interactions between perceived pitch and fundamental frequency during vowel production). These aspects depend, to a large extent, on other components of the language, namely: metrics, segmental structure, morphosyntax, semantics, information structure, and pragmatics. Therefore, the full acquisition of the prosodic system must be closely intertwined with the development of these other components (cf. Snow, 1994; Oller, 2000). Without these other components, children's predictive prosodic processing is likely to be limited.

Although children get an early start in acquiring prosodic knowledge (compared to lexicosyntactic knowledge), current evidence supports the idea that the acquisition of a full-fledged prosodic system takes many years. While certain aspects of intonational function are acquired in early infancy (e.g., speech act discrimination: Galligan, 1987; Marcos, 1987; Konopczynski, 1995; Prieto and Vanrell, 2007), others remaining elusive even for teenagers (e.g., some implications of nucleus placement and intonation grouping; Cruttenden, 1985). Intonational development has been found to correlate with grammatical development (e.g., Snow, 1994) and vocabulary size (Chen and Fikkert, 2007). Moreover, recent evidence suggests that children only process prosodic information as intonational phrases once they have acquired a certain amount of syntactic knowledge (phrasal structure; Männel and Friederici, 2010). But it may also be the case that emerging intonation is largely independent of grammatical development, at least for some children (Prieto and Vanrell, 2007).

This explanation extends to the possible predictive value of the prosodic information in our stimuli. The general finding that children's sensitivity to prosodic cues precedes their sensitivity to lexicosyntactic cues has primarily been attested in experimental tasks that tap into more localized functions of prosodic cues, and tend to focus on processing that happens below the level of the utterance (e.g., word segmentation; Nazzi et al., 1998; Grossmann et al., 2005; Christophe et al., 2008). Compared to utterance comprehension in conversational interaction, these experimental tasks operate at a different level of linguistic structure and therefore are likely to utilize somewhat different speech processing mechanisms. Prosodic comprehension in conversational contexts may be substantially different than in experimental contexts, since it is used and understood with interactive goals in mind.

Relatedly, not all prosodic cues are equally useful for predicting upcoming linguistic structure. Prosodic information in isolated linguistic forms, such as a pause or a change in pitch contour, signals a concurrent event (e.g., a syllable with a high pitch as being stressed). In contrast, prosodic information in conversation can also be used to signal *upcoming* events. More specifically, it can be used to predict upcoming prosodic phrase boundaries that can help, in turn, to pick out the intended speech act (e.g., questions vs. non-questions) and to anticipate upcoming turn structure. The use of prosodic information to make predictions in conversation requires that the listener both recognize prosodic phrase boundaries *and* map prosodic contours onto the multitude of possible pragmatic meanings. Evidently, the required linguistic knowledge that underpins the predictive use and interpretation of prosodic information becomes available to children eventually. We therefore suggest that at least some lexicosyntactic information is necessary to put prosodic information to full use in predicting upcoming turn structure during conversation (see also Männel and Friederici, 2010).

This fourth explanation also helps us to interpret the mixed evidence in prior studies about the use of prosodic information for predictive processing (Casillas and Frank, 2012, 2013; Keitel et al., 2013; Keitel and Daum, 2015). Casillas and Frank (2013) found an early, more global role of prosody in turn prediction for 1- and 2-year-olds. In their study, children's predictions only substantially improved with age for utterances with lexically-realized question markers. But, importantly, children still made the most anticipatory gaze switches when *both* prosodic and lexicosyntactic cues were available, suggesting that prosodic knowledge works together with lexicosyntactic information in predicting upcoming turn structure. In Keitel et al. s' (2013) study, only 36-month-olds were able to anticipate upcoming speaker changes and, when they did, they anticipated speaker changes better when intonation was available. Their finding is consistent with the idea that 36-month-olds use both lexicosyntactic and intonational information to predict upcoming speaker changes: 36-month-old children have acquired a substantial amount of lexicosyntactic knowledge that they can use to parse and comprehend intonation, thereby helping them to predict upcoming speaker changes. Adult controls in the same experiment also anticipated upcoming speaker changes, even without the benefit of intonation. But, because other prosodic cues were still present in the pitch-flattened stimuli, the adults in that experiment could have used alternative sources of prosodic information (final lengthening, stress and duration) to make predictions based on prosodic structure, even without intonational contours.

### Turn-projection in a more natural context

One of the goals of the study was to investigate the relative weight of lexicosyntactic and prosodic cues in full-signal speech. Prior studies have primarily used phonetic manipulation to remove lexicosyntactic (low-pass filter) and prosodic information (pitch- and duration-resynthesis Casillas and Frank, 2013; Keitel et al., 2013; Keitel and Daum, 2015). One other study used more natural speech materials to control for the presence of lexical cues, but did not control for prosody (Casillas and Frank, 2012). The current study is then the first to test the relative weight of lexicosyntactic and prosodic cues to turn transition in unfiltered, unsynthesized, and thus acoustically full, speech. The current results show that our splicing method is sufficient for investigating the use of lexicosyntactic and prosodic cues on turn prediction in both adults and toddlers.

Though we used full-signal speech, we did not aim for completely realistic stimuli. Instead, by using full-signal speech (like the speech in children's natural environment) we aimed for a balance of experimental control and increased ecological validity. Future studies could further improve the naturalness of the stimuli by making all recording stimuli in spontaneous interactive contexts, instead of pre-scripting the utterances. Read speech differs from natural speech in its prosodic properties in that it has a lower articulation rate, different pause structure (Barik, 1977), and wider pitch range (Eskénazi, 1992) than spontaneous speech. These properties are, in fact, shared with characteristics of infant-directed speech (IDS), the register that used in the present study (Fernald and Simon, 1984; Fernald et al., 1989). However, other prosodic characteristics of read speech are not common in IDS, such as fewer hesitations and fewer rising movements (Levin et al., 1982).

In sum, we showed that the relative weight of linguistic cues in toddler and adult turn projection can be investigated with relatively natural-sounding scripted conversations. Using this technique, we showed that adults and toddlers use both lexicosyntactic and prosodic cues for turn projection, but that lexicosyntactic cues are weighed over prosodic cues when the two are pitted against each other. The results present a challenge for future work to tease apart *which* lexicosyntactic cues children attend to in making their predictions, and how their use of different cues changes throughout development.

### Conflict of interest statement

The authors declare that the research was conducted in the absence of any commercial or financial relationships that could be construed as a potential conflict of interest.

## Footnotes

^1^The present study focuses on the role of intonation (one aspect of prosody) in children's prediction of upcoming speaker switches. We designed the stimuli to control for a few specific intonational contours. But, because we used a full, unfiltered linguistic signal, our intonational contours were accompanied by other prosodic cues (e.g., duration and intensity). For this reason we adopt the broader term “prosody” rather than the more narrow term “intonation.”

^2^Final-rising contours are considered prototypical for polar questions, but there are also many other intonation contours used with polar questions in spontaneous speech (see, e.g., Couper-Kuhlen, 2012).

^3^Qualtrics (2014), Provo, Utah, USA. Available from: http://www.qualtrics.com (Version 55939).

^4^The children in the current experiment are older than those in Casillas and Frank (under review; ages 1–6), so we adopted a shorter window (300ms, not 333ms) for the assumed time needed for children to plan a gaze shift (Fernald et al., 2001).
